# The importance of physical function as a clinical outcome: Assessment and enhancement

**DOI:** 10.1002/clc.23311

**Published:** 2019-12-11

**Authors:** Deirdre O'Neill, Daniel E. Forman

**Affiliations:** ^1^ University of Alberta Hospital Edmonton Alberta Canada; ^2^ Professor of Medicine University of Pittsburgh University of Pittsburgh Medical Center and VA Pittsburgh Healthcare System Pittsburgh Pennsylvania

**Keywords:** geriatric cardiology

## Abstract

The burgeoning population of older adults is intrinsically prone to cardiovascular disease (CVD) in a context of multimorbidity and geriatric syndromes. Risks include high susceptibility to functional decline, with many older adults tipping towards patterns of sedentary behavior and to downstream effects of frailty, falls, disability, poor quality of life, as well as increased morbidity and mortality even if the incident CVD was treated perfectly. While physical activity has been shown to moderate these patterns both as primary or secondary preventive medical care, the majority of older adults fail to meet physical activity recommendations. Clinicians of all specialities, including CVD medicine, can benefit from greater proficiency in functional assessments for their older adults, as well as from insights how to initiate effective functional enhancing approaches even in older adults who may be frail, deconditioned, and medically complex. Pertinent functional assessments include traditional cardiovascular metrics of cardiorespiratory fitness, as well as strength and balance. This review summarizes the components of a wide‐ranging functional assessment that can be used to enhance care for older adults with CVD, as well as interventions to improve physical function.

## 1 INTRODUCTION

It is estimated that by the year 2050, one in four Americans will be over the age of 65 years old, with the most rapid rise occurring in the subgroup >85 years.^1^, ^2^, ^3^, ^4^ Age is a prominent risk factor for cardiovascular disease (CVD) such that CVD is endemic in the burgeoning population of older adults. Older adults are not only inherently prone to CVD events, but to functional declines resulting from CVD as well as idiosyncratic age‐related vulnerabilities that typically arise as part of CVD in old age. Most older adults develop multiple CVDs simultaneously (eg, coronary artery disease in combination with heart failure and valve disease) as well as non‐CVDs (eg, chronic kidney disease, diabetes, and chronic obstructive pulmonary disease) and geriatric syndromes (eg, polypharmacy and frailty) that are conducive to compounding tendencies towards sedentariness, poor quality of life, frailty, disability, and dependency.^5^, ^6^, ^7^, ^8^ Acute disease then exacerbates the problem with hospitalization‐related deconditioning, malnutrition, sedation, and even due to many of the therapies that are utilized (eg, beta blockers). Goals to improve functional outcomes are both important as a patient's priority of care, but also as a public health mandate in a population otherwise prone to disability and high cost of care.^9^

The World Health Organization (WHO) highlights the role of function in healthy aging: “functional ability is about having the capabilities that enable all people to be and do what they have reason to value.”^10^ Regular physical activity is promoted to improve physical, mental, and cognitive function, enabling many to remain independent for a longer period of their lives.^11^, ^12^ Nonetheless, the majority of older adults are predominantly sedentary and fall short of physical activity recommendations, contributing to patterns of premature onset of illness, disease, functional decline, and mortality.^13^ Deficits in physical function have also been associated with increased risk of falls, diminished ability to live independently, and poorer self‐reported health.^14^ Thus emphasis of physical function in older adults stands out as an important opportunity to identify deficiencies, initiate solutions, and improve health.

## 2 FUNCTIONAL ASSESSMENT

Whereas there is a rich tradition of assessing cardiorespiratory fitness (CRF) as a fundamental metric of CVD health, this has ironically undercut the tendency of many clinicians to assess the broader range of functional parameters relevant to older patients. Cardiopulmonary exercise testing (CPET) presumes that a patient has requisite capacities of strength and balance that in many cases are too limited for CRF testing. Therefore, a broader range of physical function assessments is an important goal.^15^ Ideally, functional assessments for older adults need to gauge a wide range of capacities in this heterogeneous population, with prognostic sensitivity, and capacity to track changes over time (ie, sufficiently sensitive to delineate decrements or improvements).

### 2.1 Principles of cardiopulmonary exercise testing for older adults

Cardiorespiratory fitness (CRF) is a powerful indicator of morbidity and mortality. It is best measured using graded symptom‐limited CPET and directly quantified as peak oxygen uptake (VO_2_).^15^ Although standard exercise testing can be used to estimate metabolic equivalents (METs) a metabolic index conceptually related to peak VO_2_, METs are calculated and are relatively less precise than VO_2_ assessments. Peak VO_2_ declines with age, declining by 8%‐10% per decade.^16^ This is related in part to age‐related cardiovascular and pulmonary changes (eg, reduced peak heart rate), as well as to many other factors, including quality and quantity of muscle tissue, comorbidity (eg, kidney disease, diabetes, arthritis, depression), cognitive impairment, deconditioning, and even sensory changes (vision, hearing, proprioception).^17^

Exercise intolerance or dyspnea on exertion is a common clinical complaint in various disease processes. Using CPET, measurement of oxygen uptake (VO_2_), carbon dioxide exhalation, and minute ventilation in breathing during exercise (all in real time) provides a means to disentangle the relative impact of cardiovascular, pulmonary, and muscular limits on exercise performance. This can be applied to determine the dominant cause of the exercise limitation or dyspnea.^18^, ^19^ Whereas, CPET is most commonly used for disease management, particularly heart failure and pulmonary hypertension, it has potential diagnostic utility for older adults with exercise decline.^18^ Standard exercise tests provide an overall estimate of CRF in METs, whereas measurement of peak VO_2_ using CPET is relatively more accurate. Furthermore, only CPET provide diagnostic assessment of breathing limitations based on gas exchange assessments.

The choice of exercise modality and the intensity of exercise testing provocation are usually key determinants of a CPET's efficacy. Both treadmill and bicycle modalities are common options. Treadmills enable walking protocols in older adults, an activity that is usually relatively more familiar and comfortable to many, and which also entail more muscle mass (compared to seated cycling) which adds to the physiological value of testing. Nonetheless, bicycle protocols are still preferred by many testers and patients as they may seem relatively more secure and/or achievable for those struggling with frailty, deconditioning, dizziness, falls, orthopedic limitations, or anxiety.

Many exercise testing labs default to the Bruce treadmill protocol (a treadmill protocol that entails the rapid progression of speeds and inclines over a few minutes to demarcate exercise limitations). Similarly, many use high rapid progressions of resistances for their bicycle testing (25 watts/min).^15^ Rationale to use these protocols is based on their diagnostic and prognostic efficacy in younger populations.^15^ Unfortunately, these protocols rarely work well in older populations, and older patients who may be relatively weaker, deconditioned, and infirmed can become overwhelmed with these progressions. Instead, exercise testing protocols with gentler progressions of inclines, speeds, and/or bicycling resistance are usually much more effective. The “modified Bruce protocol” starts at a lower workload and is generally better tolerated by patients who are sedentary or deconditioned. The Naughton and Balke protocols are similarly low‐intensity protocols, and are particularly useful for those with limited CRF.^15^, ^20^ Regardless of the modality and protocol used, the overall goal is to individualize the exercise provocation to enable an assessment of peak capacity over about 10 minutes.^15^

### 2.2 Broader perspective of physical assessment

Beyond the traditional models of stress testing, better assessments of strength, balance, and activity have particular relevance in an older population.^8^

1. The 6‐minute walk test (6MWT) is a sub‐maximal exercise test commonly used to assess function and functional capacity.^21^ In higher functioning adults, 6MWT has been associated with CRF, but other more fundamental physiological limitations may also undercut performance during a 6MWT. While there are often site‐specific aspects to the 6MWT assessment, a 30‐m course is generally recommended, with chairs are set up at each end as well as halfway along this course. Patients are instructed to walk back and forth as fast as possible over 6 minutes. Patients are told then can slow down, stop, and sit as necessary, to then resume walking when they are able, thereby generating a result for every patient.^22^, ^23^ Whereas a so‐called “normal” 6‐minute walk distance in healthy adults is reported from 400 to 700 m, many older community‐dwelling can only walk 200 to 300 m, reflecting changes in CRF as well as in strength and balance, and related changes in posture, muscle, autonomic function, vision, and other decrements of age. Age, height, and sex‐specific reference values are available to interpret 6MWT scores.^24^, ^25^
2. Gait speed is a valuable tool to gauge functional status and physiological reserve in a manner that is independent to stress testing or 6MWT, particularly as it is relatively more independent of CRF physiology.^26^, ^27^ A gait speed assessment is also relatively easier to perform than walking for 6 minutes for many old frail patients. While many variations on gait speed assessments have been utilized, a well‐validated protocol entails a 4‐m course from a standing start.^28^ Patients are instructed to walk at their normal pace, using an assistive device if needed. The patient is timed and their gait speed is calculated. A gait speed of 1.4 m/s indicates the patient is more likely to be independent in ADLs, whereas a gait speed of <0.8 m/s suggests the patient is more likely to be frail, have increased fall risk and have functional dependency in ADLs and IADLs.^29^
3. The short physical performance battery test (SPPB) is one of the most commonly used and validated measures of physical function. It is a more comprehensive assessment of physical function, combining assessments of gait speed, balance, and strength. A 4‐m gait speed is measured from rest (as described above). Balance is measured with several foot positions, including tandem stand and semi‐tandem stand. Strength is measured as the time required to complete five chair rises. A maximum score of 12 is achieved by healthy people, with a score of ≤10 predicting physical disability in mobility and a score of ≤6 predicting fall risk and ADL disability.^23^, ^30^ Guralnik et al developed the SPPB, using it in a cohort of individuals >70 years of age without baseline ADL disability, following them over time. Those with lower SPPB scores at baseline were four to five times as likely to develop ADL disability during the 4‐year follow up.^31^
4. The timed up and go test (TUG) is also a well‐validated and frequently‐used functional assessment found to be predictive of fall risk.^32^ The test involves a patient starting in the seated position, getting up without the use of the arms of a chair, walking a pre‐measured distance of 3 m and returning to the seated position.^32^ A normal result is 8 to 12 seconds, with a cut‐off score of >12 seconds indicating increased fall risk.^23^, ^33^
5. *Activities of daily living*: An initial assessment of function also entails evaluating the patient's current functional status, identifying existing impairments in basic activities of daily living (ADL) and instrumental activities of daily living (IADL). Basic activities of daily living are skills need to manage one's basic physical needs, or self‐care activities, including bathing, grooming, toileting, transferring, and feeding. Instrumental activities of daily living are more complex activities necessary for independent living in the community—such as cooking, cleaning, shopping, taking medications, managing finances, and using the telephone. This can be assessed by self‐report, proxy/caregiver reports or direct observation, using performance‐based measures such as the Katz Index of Independence in ADLs or Barthel ADL Index, more commonly administered by occupational therapists.^34^ It is estimated that over 22% of those over age 75 years and over 40% of those over 85 years struggle to perform their basic ADLs.^35^ Additionally, ADL impairment is a strong predictor of outcome, predicting longer length of stay in hospital, functional decline, increased risk of institutionalization and death, thus important to assess.^14^, ^36^
6. *Cognitive function*: A brief screening for cognitive impairment is another important component of the assessment of function as cognition has enormous bearing on physical activity, exercise, as well as activities of daily living.^34^ The prevalence of cognitive impairment increases with age, and is especially high in those with CVD, yet it often goes undiagnosed, as many adults with cognitive decline remain community dwelling but simply restrict their lives, and/or become more dependent on those around them. The literature supports use of a rapid dementia screening test, such as the Mini‐Cog, as a quick and easy assessment of cognitive changes. The Mini‐Cog is a 3‐minute instrument, consisting of a three‐item recall test for memory and a clock drawing test.^37^ The patient receives 1 point for each word recalled and 2 points for a normal clock or 0 points for an abnormal clock. A total score of 3, 4, or 5 indicates a low likelihood of dementia, whereas a lower score suggests some degree of cognitive impairment and the patient should be referred for further assessment.
7. *Depression*: Assessment of depression is also relevant to functional evaluation in an older adult with CVD. Depression is highly prevalent (estimated to be 13% in those aged 80 years and older) and can result in significant functional impairment (in part due to related effects on cognition), and it is treatable.^38^ A number of screening tools exist to detect depression in older adults, but one of the more commonly used is the Geriatric Depression Scale (GDS). The short form GDS consists of 15 questions and takes approximately 5 to 7 minutes to complete. A score of 0‐4 is normal, 5‐8 suggests mild depression, 9‐11 moderate depression and 12‐15 indicates severe depression.^39^ The presence of depression mandates prompt referral for appropriate intervention and treatment.

## 3 IMPROVING FUNCTIONAL CAPACITY

Functional capabilities in older adults have significant bearing on health and well‐being. High physical function in earlier phases of old age (65‐80 years), has been correlated to functional independence that extends well beyond 80 years.^40^ A decline in CRF, strength, or balance indicates that everyday activities entail a greater proportion of an older adult's functional reserve. Climbing stairs may no longer be feasible if it entails more workload or capacity than an older adult can achieve. People can become trapped in their chairs, no longer able to stand, much less walk to the bathroom or grocery store. These are common scenarios, with many older adults, particularly those with CVD, susceptible to progressive sedentary behavior and functional decline. In addition, older adults commonly have more numerous accumulated comorbidities, further increasing vulnerability to becoming sedentary, resulting in poorer baseline function and less reserve if hospitalization is to occur. This also results in a higher likelihood of development of disability, dependency, and frailty.^17^

### 3.1 Lifelong habits and recovering from CVD

Arterial compliance is known to change with age, with changes including reduced compliance, impaired vascular endothelial function and increased intima‐media wall thickness, particularly in older adults who are sedentary.^41^ Habitual physical activity has been shown to reduce the risk of CVD and result in less or no age‐related decrease in arterial compliance compared to sedentary peers.^41^, ^42^ Additionally, short‐term exercise interventions in sedentary adults have been shown to improve vascular compliance and endothelial function.^41^, ^43^ However, the degree of benefit may vary in relation to the types of type of exercise and other differences in diet, environment, and health.^41^, ^44^, ^45^, ^46^, ^47^ In general, regular exercise is recommended to mitigate much of the otherwise predictable age‐related vascular changes that predispose to CVD and other vulnerabilities of aging.

Whereas many types of physical function are important, the physiological implications of aerobic training (with the goal to improve CRF) has been the most substantively supported, with decades of research demonstrating its correlation to cellular respiration and integrated physiological health. Lower CRF has been shown to be a significant determinant of functional dependence in an 8‐year follow‐up study of older adults.^48^

Related work has demonstrated that low CRF can be modified. Usually it is assumed that CRF relates primarily to aerobic activities, but this presumes that adults have sufficient strength and balance for aerobic activities to be feasible. Boyle et al found when more than 1000 older adults, mean age 80.4 years, performed an average of 3 hours per week of physical activity, the risk of developing ADL or IADL disability decreased by 7% for each additional hour of physical activity performed per week (HR 0.93, 95% CI 0.88‐0.98 for ADL and HR 0.93, 95% CI 0.89‐0.99 for IADL).^49^ A systematic review found consistent evidence to support community‐dwelling older adults participation in regular aerobic activity reduced the risk of functional limitation and disability.^11^ Although the exact exercise recommendation is unclear, it appears moderate to higher levels of activity are more effective for the most significant impact on functional outcomes.

Despite evidence supporting the functional benefits of routine aerobic exercise, inactivity is highly prevalent, especially in the aging population. The Centers for Disease Control (CDC) reported that the prevalence of inactivity is 25% in those 50‐64 years of age, 27% in those 65‐74 years and 35% in those over age 75 years.^13^ Non‐Caucasians, women, those with lower education levels, increased BMI and with one or more chronic diseases are even more likely to be inactive. Physicians and health care professionals can be influential in changing the behavior of sedentary older adults, with exercise counseling by a primary care physician being shown to increase patients' participation in physical activity. Weidinger et al found patients who saw a doctor regularly were 54% more likely to be physically active and they were nearly five times more likely to meet physical activity recommendations if their doctor helped them develop an exercise plan (adjusted OR 4.99, 95% CI 1.69‐14.73).^50^ The same is true for participation in cardiac rehabilitation (CR), with the American Heart Association publishing a scientific advisory in 2012 on the importance of healthcare professionals' role in the promotion of CR, acknowledging the valuable role of the healthcare professional in bridging the gap between inpatient care and participation in outpatient CR.^51^

Cardiac rehabilitation provides a safe and effective means to re‐introduce vital aerobic activity and augmentation of CRF. Cardiac rehabilitation is a multidimensional and comprehensive treatment program, typically involving medical evaluation, exercise training, cardiac risk factor modification and education, aimed to promote lifelong health and wellness in those with CVD.^52^, ^53^ Functional improvements achieved in CR have also been demonstrated to enable older adults to perform activities of daily living at a lower percentage of overall CRF such that efficiency and confidence are improved.^6^ As it is now well‐known that adults with CVD are more likely to attend CR when healthcare providers make the recommendation in person,^54^ communication regarding the importance of CR (and activity in general) is an essential part of optimal care.

### 3.2 Strength training

Age‐related loss of muscle mass, strength, and function is manifest in both sarcopenia and related susceptibility to frailty.^55^, ^56^ Evidence has shown that older adults lose approximately 0.5% of their total skeletal muscle mass per day and 0.3% to 4.2% of their muscle strength per day.^57^ Weakening attributable to muscle atrophy is compounded by changes in intrinsic muscle quality, such that the remaining muscle provides less force and efficiency. Causes of muscle changes are multifactorial, and include sedentariness, insufficient dietary intake, reduced type II skeletal muscle fibers, and reduced insulin‐like growth factor 1.^58^ Reduced strength is an important determinant of overall function in older adults, and is predictive of future functional decline and higher incidence of frailty, disability, and mortality.^59^, ^60^

Strength or resistance training has been shown to increase muscle mass, muscle strength, and protein synthesis.^58^ A study by Chen et al randomly assigned older adults aged 65‐75 with sarcopenic obesity to resistance training, aerobic training, combination training, or no intervention, over an 8‐week period. Muscle strength was found to be higher in the resistance training group compared to all other groups at 8 and 12 weeks.^58^ When resistance training was compared to a control intervention of yoga or breathing exercises in women ≥65 years old with CVD, the 6‐month resistance training program resulted in statistically significant improvement in physical work capacity, balance coordination, and 6MWT performance, as compared to the control intervention.^61^

Even very old patients with baselines of very poor function benefit from strength training. Fiatarone et al showed that a several week strength training program for institutionalized octogenarians and nonagenarians increased strength substantially, with many individuals being able to reduce their dependence on wheelchairs and walkers.^62^ Therefore, resistance training can help prevent and treat sarcopenia as well as promote beneficial effects in strength and endurance. Consistently, for many older adults, increasing strength is the critical first step towards capacity to enable aerobic training and improving CRF. Furthermore, strength training enhances muscle power and maximal neuromuscular activity, helping to improve function, and modify risk of falls.^63^

It is known that caloric intake declines with increasing age, further exacerbated by the appetite changes, and decreased hunger sensation occurring with age.^64^, ^65^, ^66^ This reduced energy intake can lead to inadequate protein, vitamin, and mineral intake, and resulting clinical or subclinical nutritional deficiencies which can adversely affect health and function.^67^ Sarcopenia and frailty often co‐exist with malnutrition in older adults.^68^, ^69^ Proper nutritional supplementation may be able to augment functional decline, thereby delaying or preventing functional disability. Research is ongoing into the importance of nutrition in muscle mass, strength and function in older adults. Currently, the strongest evidence exists for dietary protein in addition to physical activity in muscle protein synthesis.^64^ Low protein intake has been linked to loss of muscle mass and strength in older age,^70^, ^71^, ^72^ with suggestion that higher protein intake is associated with preserved muscle strength.^72^ However studies on the effect of protein supplementation on function show mixed results and thus, research is ongoing.

Over a third of older adults over the age of 65 years in the United States are obese, with obesity worsening age‐related functional decline and frailty.^73^, ^74^ There has been concern that weight loss in older adults may inadvertently result in increased risk of malnutrition and sarcopenia. This was studied by Villareal et al, who randomly assigned 160 obese older adults, aged 65 years and older, to weight management plus aerobic training, resistance training or combination aerobic and resistance training, or the control group of weight management without exercise, with the primary outcome of change in the physical performance test score.^75^ Physical performance increased the most when weight loss was combined with aerobic and resistance training, but all the exercise arms experienced a greater improvement in physical performance than the control group without exercise (*P* < .001 for all comparisons). This has been further investigated at the cellular level by Colleluori et al, who found that in obese older adults undergoing weight loss, combination aerobic, and resistance exercise resulted in superior muscle synthesis and muscle quality than either mode of exercise alone, after comparing muscle biopsies at baseline and 6 months.^76^

### 3.3 Balance training

Balance, while rarely a concern for younger adults, is a critical issue for many older adults, with imbalance resulting primarily from muscle weakness and further exacerbated by vasoactive medications, reduced thirst sensation, neuropathy, and impaired vision. Balance is important as it impacts gait, increases the risk of fall and injury, and worsening balance has been shown to correlate with lowered functional independence.^77^ It is estimated that one in every three adults ≥65 years of age, and close to half of those ≥80 years of age suffer at least one fall annually.^78^ Sustaining a fall, even without serious injury, has been shown to result in function decline, poorer self‐rated health, and fear of falling that can impair ADL ability and negatively affect one's quality of life.^79^, ^80^

Exercise training has been shown to improve balance, particularly the combination of balance and strength training. Improvements in postural control and maintenance of upright stance, and reduced incidence of falls have been demonstrated.^81^, ^82^ Barnett et al studied 163 adults ≥65 years old, who after screening, were at risk of falling.^81^ Patients were randomized to an exercise intervention or control. At baseline, groups were similar in terms of physical performance, health, and activity level. The exercise group had a 40% lower rate of falling than that of the control group (RR 0.60, 95% CI 0.36‐0.99). Weerdensteyn et al studied 113 older adults with a history of falls, and showed that exercise decreased falls by 46% (IRR 0.54, 95% CI 0.34‐0.86) compared to the control group, and balance confidence scores were improved in the exercise group.^82^

Tai Chi is a widely adopted form of low intensity exercise with integrated components of strength, balance, and aerobic training and may be particularly helpful in balance retraining and fall prevention. A study of Tai Chi compared to a 6‐month stretching program found that Tai Chi resulted in fewer falls, fewer injurious falls, improved balance, and reduced fear of falling, as compared to those in the stretching group.^83^ Additionally, Tai Chi improved CRF in older adults, resulting in less decline in peak VO_2_,^84^ improved ADL performance in Parkinson's disease,^85^ and improved IADLs in older adults with mild cognitive impairment.^86^

## 4 CARDIAC REHABILITATION

Cardiac rehabilitation has traditionally been associated with aerobic training and related goals to improve CRF, but it is a multi‐composite program, providing opportunities to address strength and balance, as well as aerobic training goals for older patients with CVD, including the many who are frail and deconditioned. Similarly, it provides opportunities to address interrelated challenges of nutrition, mood, cognition, and other age‐related challenges that commonly confound CVD management in older adults. Despite outpatient phase II CR being a class I recommendation from the American Heart Association and American College of Cardiology after MI, coronary revascularization, valvular heart surgery, post‐heart or heart‐lung transplant, and heart failure with reduced ejection fraction, only between 13% and 34% of eligible patients attend.^17^, ^87^ The remarkably low enrollment and participation rates are multifactorial. Transportation is a prominent barrier to many. Attending facility‐based CR is also difficult when the participant is a caregiver to an infirmed spouse or if they assist in childcare of grandchildren. Socioeconomic stress is also pertinent as many older adults live on a fixed income, making the co‐pay required to participate in CR a substantial barrier.

The evolving implementation of home‐based CR may ultimately respond to this clinical challenge, but safety and efficacy of home‐based CR remains to be refined for medically and physically complex older frail patients. Similarly, the potential of wearable technologies and/or portable devices to deliver and track CR is evolving, but the applicability to very old frail adults remain to be fully refined and proven.

## 5 OTHER BARRIERS TO EXERCISE AND PHYSICAL ACTIVITY

Socioeconomic, cultural, and environmental factors are significant barriers to exercise and physical activity for older adults.^88^ As mentioned above, many older adults no longer drive and lack alternative transportation. This becomes an even more difficult challenge in colder months, as older adults have a higher risk of falls, injury, and even hypothermia related to the weather.^89^ Additionally, older adults are more often on a fixed income, making the commitment of a gym/recreation center membership a financial strain. Many also have caregiving responsibilities to an even more infirmed spouse. Therefore, goals to overcome sedentariness demand a holistic perspective.

Although exercise throughout a person's lifespan is ideal, the introduction of exercise in late‐life has been shown to be beneficial to functional independence in later‐life. Stessman et al studied the effect of regular exercise activities in older adults on independence and ADL performance and found that exercise 4 days per week at age 70 resulted in increased ease of independent ADL performance at age 77 years.^90^ Berk et al found inactive older adults who increased their exercise achieved similar improvements in disability as compared to participants who were more active throughout the 16‐year longitudinal study,^91^ suggesting exercise is beneficial to preservation of function, even when introduced in later life.

## 6 IMPROVING COGNITION WITH EXERCISE AND PHYSICAL ACTIVITY

Cognitive impairment is common in older adults and is a significant predictor of functional decline and disability, limiting the independence of older adults.^92^ There is suggestion that exercise interventions may improve cognitive function. A systematic review and meta‐analysis of 39 RCTs on exercise interventions in community dwelling adults ≥50 years found that exercise significantly improved cognitive function (*P* < .01), with aerobic training, resistance training, multicomponent training, and tai chi all having significant point estimates.^93^ They found that 45 to 60 minutes of at least moderate intensity exercise was most beneficial to cognitive function and the effect was independent of baseline cognitive status. Longitudinal studies suggest older sedentary adults who maintain an exercise program for at least 6 months perform better on measures on processing speed, executive function, and memory.^94^ A meta‐analysis by Gheysen et al found that physical activity combined with cognitive activities resulted in significantly larger gains in cognition as compared to physical activity alone, suggesting a combination of physical activity and cognitive exercises are perhaps the most effective modality for preventing cognitive decline in older adults.^95^

## 7 MODIFYING FRAILTY WITH EXERCISE AND PHYSICAL ACTIVITY

Frailty is a syndrome of decreased reserve and vulnerability to stressors, resulting from cumulative declines across various physiologic systems. Older adults who are frail or pre‐frail are at an increased risk of functional dependence and ADL disability,^96^ highlighting the importance of interventions to reduce this risk. Whereas frail patients are often regarded as inappropriate for physical activity programs or rehabilitation because they are frail, they may paradoxically have the most to gain. In a seminal study, Fiatarone et al published a randomized control trial of 100 frail nursing home residents, mean age 87 years old, randomized to a progressive resistance exercise training program, multi‐nutrient supplementation, both or neither intervention, over a 10‐week period.^97^ Those receiving the exercise intervention demonstrated increased muscle strength, gait velocity, stair climbing power, and cross‐sectional thigh muscle area, as compared to those who did not exercise, showing that even in oldest old, frail, nursing home residents, a short 10‐week exercise program resulted in significant improvement in physical function and frailty. Another study randomized 115 older adults, mean age 83 years, with mild to moderate frailty, to a low‐intensity flexibility‐focused home exercise program or a progressive exercise training program.^98^ The exercise training group improved significantly in three of four measures of physical function, again demonstrating that intensive exercise training can improve physical function in very old adults with baseline frailty and disability.

A recent systematic review of randomized controlled trials on the best intervention for frail and pre‐frail older adults found physical activity is key in the maintenance of functional independence.^99^ Besides physical activity, some evidence exists for the utility of nutritional enhancement, particularly protein supplementation for patients with muscle atrophy, however at this time, the utility of nutritional supplementation remains a dynamic area of investigation.^100^

### 7.1 Improving quality of life with exercise and physical activity

Quality of life is an outcome important to most older adults and is intimately related to ability to function and independence. Fusco et al showed that in a group of older adults, mean age 77.6 years, women perceived any deterioration in IADL disability as significantly worsening their QOL.^101^ ADL function has been shown to have bearing on quality of life throughout the lifespan, including for adults living in a nursing home.^102^ Life satisfaction is a construct similar to QOL, and has also been shown to be contingent on functional capacity.^103^, ^104^

Literature suggests older adults can improve QOL by engaging in physical activity. When targeting improved QOL with exercise however, it is important that the exercise prescription for the older adult focuses on an area of function important to the individual and relevant to their daily lives.^105^ This information may be determined by way of motivational interviewing^106^ and is being explored further in the setting of CR in a recently initiated NIA trial, Modified Approach to Cardiac Rehabilitation in Older Adults (MACRO) (http://clinicaltrials.gov: NCT03922529NIH).

A rich literature demonstrates the links between QOL and physical activity. Yen and Lin introduced physical activity to older adults in a long‐term care facility in Taiwan, showing significant QOL benefits.^107^ Lavie and Milani compared older adults ≥70 years of age to younger patients <55 years in CR, finding those ≥70 years had significant improvement in QOL scores, even greater than in younger patients (20% vs 15%, *P* = .03).^108^ Another study by Lavie and Milani showed QOL, pain, energy, physical function, well‐being, general health, and mental health scores all significantly improved in cardiac patients who participated in CR, with similar benefits in patients ≥65 years and younger cohorts.^109^

### 7.2 Reducing depression with exercise and physical activity

Depression is prevalent in older adults, with an estimated 34‐million Americans ≥65 suffering from depression.^110^ It is also highly prevalent in patients with CVD, with estimates that depression in those with CVD occurs two to three times more often than in the general public.^111^ Furthermore, depression correlates to a 4‐fold increase in death post‐MI, as well as increased healthcare utilization and decreased perceived quality of life.^112^, ^113^ Depression is also related to declining physical function. The aging and longevity study iISIRENTE, studied community‐dwelling older adults aged 80 years and older, and showed that depressed participants were more functionally impaired, including higher number of impairments in IADLs.^114^ Lower physical function was also associated with increased depression and anxiety, even in those with cognitive impairments. A study of 170 nursing home residents, aged 60 to 100 years, with mild to moderate dementia showed individuals with better muscle strength, balance and faster walking speed, therefore better functional ability, had less depressive symptoms than those with worse functional status.^115^

Treatment of depression can lead to improved physical function. Callahan et al showed older patients with depression who were randomized to a collaborative treatment intervention for depression, not only benefitted with reduced depression, but also demonstrated improved physical function.^116^ Evidence suggests that in patients with CVD and depression, CR plays a valuable role, with reduced prevalence of and symptoms of depression by 50% to 70%.^117^ Milani et al studied 522 consecutive CVD patients, mean age 64 years, and showed that prevalence of depression decreased 63% after CR, from 17% to 6% (*P* < .0001).^117^ Furthermore, those who remained depressed after CR had a greater than 4‐fold higher mortality compared to those who were no longer depressed (22% vs 5%, *P* = .0004) and those who were depressed but completed CR had a 73% lower mortality than control patients who were depressed and did not complete a CR (8% vs 30%, *P* = .0005). Even mild improvements in fitness level were associated with decreased depression and mortality. A meta‐analysis of 18 randomized controlled trials assessed the effect of CR on depression in older adults, age > 64 years, showed exercise combined with psychological interventions was more effective in decreasing depression than usual care.^118^

### 7.3 Improving self‐efficacy with exercise and physical activity

Self‐efficacy is defined by Bandura as a patient's belief that they have the ability to influence their lives via self‐imposed actions.^119^ Higher self‐efficacy has been related to higher self‐esteem, increased quality of life, increased ADL participation, reduced depression and anxiety, and better disease management.^120^ A study of 94 healthy, inactive older adults, mean age 73 years, randomly assigned to twice weekly Tai Chi or control found that Tai Chi resulted in improved self‐efficacy, which related to increased levels of perceived physical capability, and improved physical function.^121^ Older patients undergoing rehabilitation post‐stroke were assessed in terms of fall‐related self‐efficacy and its relationship to function.^122^ Patients with lower self‐efficacy had less improvement in function than those with high self‐efficacy, suggesting again the importance of improving self‐efficacy in order to enhance physical function.

To enhance self‐efficacy and thereby physical function, exercise is beneficial. A randomized controlled trial of 174 older adults, aged 60 to 75 years, compared a 12‐month exercise program to a stretching/toning control program, finding significant increases in all levels of self‐esteem in the exercise intervention group.^123^ Another trial of over 400 older adults aged 70‐89, all at risk for disability, showed the value of a 12‐month exercise intervention.^124^ Even in very old adults with baseline functional impairments, the exercise training intervention resulted in greater favorable changes in self‐efficacy and physical functioning than controls.

## 8 CONCLUSION

Functional limitations are a predictable part of CVD disease, with substantial impact on CVD outcomes. Multiple factors contribute to the vulnerability of older adults with CVD to functional declines, as well as the difficulty to modify these patterns. It is important that clinicians are proficient in functional assessment as part of CVD management and that functional decrements are addressed as part of routine care. Overall, physical activity has been shown to improve CRF, muscle strength, balance, cognition, depression, and self‐efficacy, all resulting in improved ADL and IADL capability and functional independence. This translates in both the efficacy and patient perceived value of care.
